# Combining Charge Couple Devices and Rate Sensors for the Feedforward Control System of a Charge Coupled Device Tracking Loop

**DOI:** 10.3390/s16070968

**Published:** 2016-06-25

**Authors:** Tao Tang, Jing Tian, Daijun Zhong, Chengyu Fu

**Affiliations:** 1 Institute of Optics and Electronics, Chinese Academy of Science, Chengdu 610209, China; abb1978@163.com (J.T.); zqfirefly@126.com (D.Z.); cyfu@ioe.ac.cn (C.F.); 2 Key Laboratory of Optical Engineering, Chinese Academy of Sciences, Chengdu 610209, China; 3 University of Chinese Academy of Sciences, Beijing 100039, China

**Keywords:** sensor fusion, charge couple device, feedforward control, time delay, light of sight error, FOG

## Abstract

A rate feed forward control-based sensor fusion is proposed to improve the closed-loop performance for a charge couple device (CCD) tracking loop. The target trajectory is recovered by combining line of sight (LOS) errors from the CCD and the angular rate from a fiber-optic gyroscope (FOG). A Kalman filter based on the Singer acceleration model utilizes the reconstructive target trajectory to estimate the target velocity. Different from classical feed forward control, additive feedback loops are inevitably added to the original control loops due to the fact some closed-loop information is used. The transfer function of the Kalman filter in the frequency domain is built for analyzing the closed loop stability. The bandwidth of the Kalman filter is the major factor affecting the control stability and close-loop performance. Both simulations and experiments are provided to demonstrate the benefits of the proposed algorithm.

## 1. Introduction

A direct feedback loop is usually utilized to control LOS in a CCD-based tracking system [1,2,3]. High control bandwidth facilitates better closed loop performance. However, limited sampling frequency and time delay are the major reasons to restrict the bandwidth. Time delays, namely including exposure time of the CCD, image process time and transmit time, cannot be cut to zero, resulting in ineffectiveness of the high bandwidth. The Smith predictor is introduced into the closed-loop system to compensate for time delays [4]. Experiments verify rate feed forward control to effectively improve the tracking performance, especially for a maneuvering target tracking [2,5]. How to obtain LOS rate is a major task to implement a feed forward control, because a tracker such as CCD cannot provide target trajectories or even target velocities but only the target error. LOS rate estimation usually synthesizes LOS error, encoder and rangefinder in a stationary platform [6]. An inertial measurement unit is required if estimating the LOS rate on an inertial stabilization platform [7]. Only employing LOS error and gimbal position was developed to generate LOS rate for compensation of LOS error [8]. As far as LOS rate estimators are concerned, there are many researchers who have developed new methods although they could not be used for feed forward control [9,10,11]. The Kalman filter is used to implement these estimation algorithms, especially for tracking a maneuvering target. Some papers focus on optimizing the Kalman filter to adapt to maneuvering targets [12,13,14,15]. However, the closed-loop stability is not taken into account when the LOS rate is not available. This paper proposes combining the CCD and FOG to recover the target trajectory as observed values of a Kalman filter which can produce the LOS rate to implement feed forward control. Additive feedback loops are inevitably added to the original control loops because some closed-loop information is utilized. The closed-loop stability and robustness are investigated on the condition of gain margin and phase margin of the open-loop transfer function with feed forward control. To analyze the closed-loop stability, the transfer function of the Kalman filter in the frequency domain is built.

Section 2 presents a detailed introduction to feed forward control based on sensor fusion, mainly describing the implementation of feed forward control; Section 3 focuses on parameter design, to be specific in terms of tracking controller and the Kalman filter; Section 4 discusses and analyzes system stability and sensitivity function; Section 5 sets up simulations and experiments to testify the theorems above; concluding remarks are presented in Section 6.

## 2. Feedforward Control Based on Sensor Fusion

**Figure 1:**
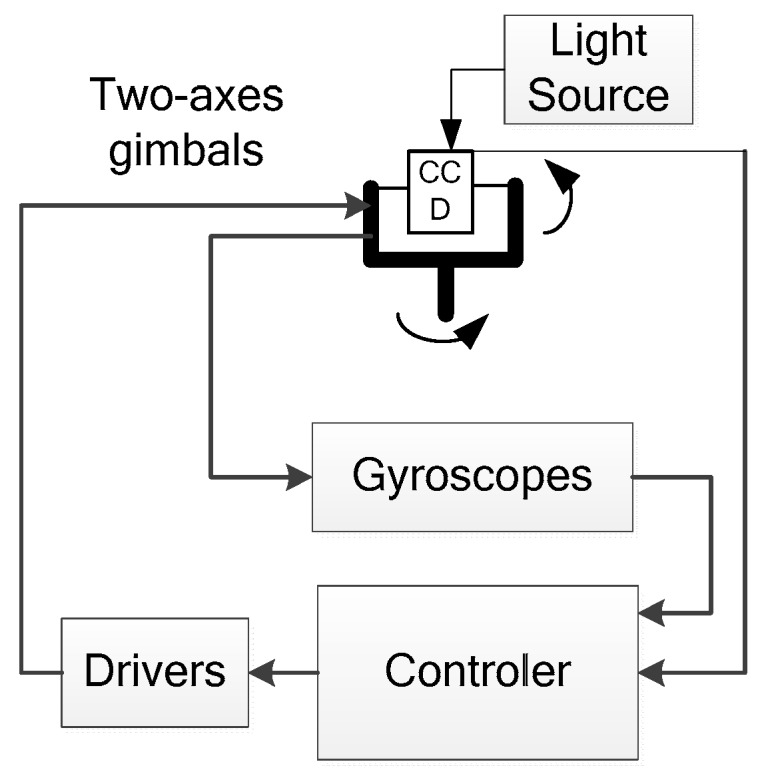
Configuration of the tracking control system.

The configuration of the CCD-based tracking system is a two-axes gimbals design illustrated in Figure 1. The sensors include FOG and a CCD. The FOG gyroscope mounted on the gimbal is usually used as the feedback component of the velocity closed loop. The controller is used to implement the control algorithm. The driver actuates the motors to achieve the tracking control. The light source is used to simulate the target of the CCD.

**Figure 2:**
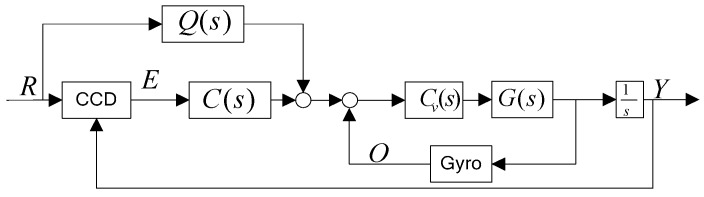
Classical feed forward control.

The control mode of Figure 1, which includes two closed loops (position loop and velocity loop) and feed forward loop is shown in Figure 2. *Q*(*s*) is the feedforward controller. *G*(*s*) is the control plant. *C*(*s*), *C_v_*(*s*) are the position controller and the velocity controller. The time delay $e^{-T_{0}s}$ characterizes the CCD in the control system although it may be rough. R represents the target trajectory. E is the line of sight error. O is the output of the fiber-optic gyroscope (FOG), which provides the gimbal rate. A FOG providing the angular velocity of the gimbal usually has high bandwidth, resulting in a little effect towards to the closed-loop bandwidth. Therefore, the characteristic of the gyro can be considered to constant one in this control system.

The perfect feed forward control requires *Q*(*s*) = *sP*^–1^(*s*).The time delay cannot be compensated, so the perfect controller is expected to implement *Q*(*s*) ≈ *sP*^–1^(*s*). *P*(*s*) called the velocity closed-loop transfer function and is defined as follows:

(1) $$P(s)=\frac{C_{v}(s)G(s)}{1+C_{v}(s)G(s)}$$

As a matter of fact, this term $\frac{C_{v}(s)G(s)}{1+C_{v}(s)G(s)}$ is very close to constant at low frequencies because the velocity closed loop has much higher bandwidth than that of the position closed loop. Thus, *P*(*s*) ≈ 1 is true to some extent. However, it is still impractical to implement *Q*(*s*) = *sP*^–1^(*s*), because the term *P*(*s*) includes not only non-nominal part, but also high-frequency characteristics. In this case, the feedforward controller can be described as:

(2) $$Q(s)=\frac{s}{1+T_{f}s}$$

The phase lag term 1/(1 + *T_f_s*) indicates the main feature of a filter.

**Figure 3:**
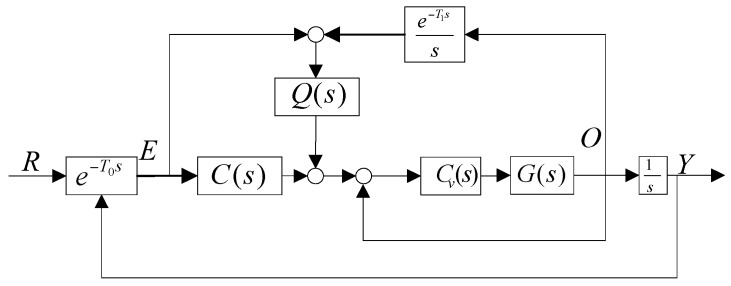
An equivalent feed forward control.

In a CCD-based control system, the CCD only provides LOS error while R is not available, so an equivalent control structure of Figure 2 is depicted in Figure 3.

This control structure in Figure 3 is practical. This equivalent feed forward control combines the the CCD and rate sensor to recover the target trajectory produce the LOS rate to implement feed forward control. From Figure 3, we have:

(3) $$R=E+\frac{e^{-T_{1}s}}{s}O$$

The time delay *e*^–*T*1*s*^ is used to match the feature of delay about CCD. It is impossible to set *T*_1_ = *T*_0_, because the time delay of CCD is uncertain although the sample frequency of FOG can reach several thousand Hertz or more, while the CCD usually has a frequency of dozens of Hz. Differentiating the synthesizing signal $E+\frac{e^{-T_{1}s}}{s}O$ produces the line of sight rate due to bad noise, resulting in ineffectiveness. Therefore, a Kalman filter is used to estimate the line of sight rate in this paper. Before this, how the realized R as the observed value of the Kalman filter is the first step.

**Figure 4:**
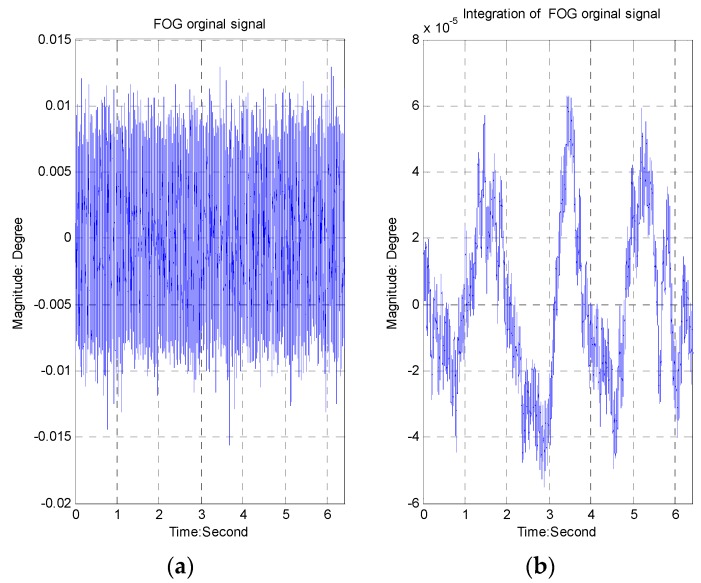
FOG Noise Characteristics FOG Noise Characteristics (**a**) The FOG original signal; (**b**) The integration signal of the FOG.

The bandwidth of the FOG is above 500 Hz. The characteristics of the FOG noise are depicted in Figure 4 (Figure 4a represents the original signal, while Figure 4b describes the integration signal). The peak value of the FOG noise is equal to 0.025°/s, and the RMS value is about 0.0037°/s.

**Figure 5:**
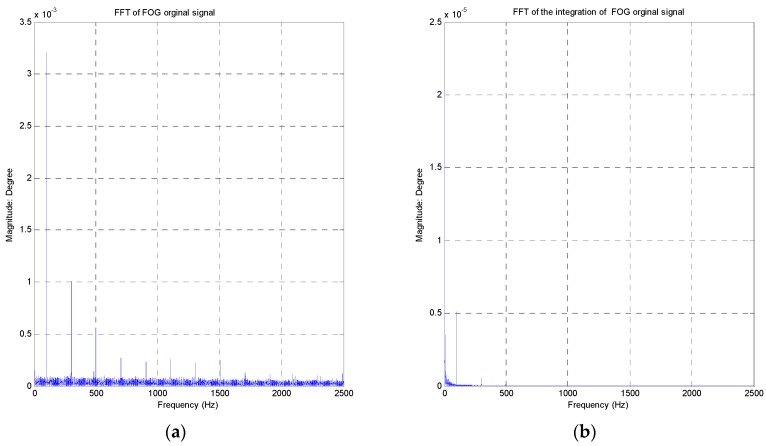
FOG FFT analysis (**a**) FFT of the original signal; (**b**) FFT of the integration signal.

Besides some “spikes”, the amplitude value of amplitude-frequency curve is smooth in Figure 5 (Figure 5a represents the FFT of the original signal, while Figure 5b describes the FFT of the integration signal). Especially, the integration of FOG signal is smoother due to the integral impact.

The CCD characteristic in this system is shown in Table 1.

The maximum focus length is 450 mm, so the instantaneous field of vision is about 12.2u rad.

The integration of FOG signal is depicted as follows:

(4) Int_FOG(i+1)=O*Δt+Int_FOG(i), i=0,1,2,3⋯

where Δt is the sampling time of CCD. Therefore, the precision of the integration of FOG is about 0.02 × 0.025 = 0.0005°, smaller than 12.2u rad. Combining Equations (3) and (4), we have:

R=E+Int_FOG(i+1)

## 3. Parameters Design

The Kalman filter is employed to implement the filter *Q*(*s*), because it is an optimal LMS filter to suppress noise [12]. The standard Kalman equations are depicted as follows:

(5) $$\left\{ \begin{array}{l} x_{k+1}=Ax_{k}+Bu+w_{k}\\ y_{k+1}=Cx_{k}+v_{k} \end{array}\right.$$

The observed value of Kalman filter is $E+\frac{e^{-T_{1}s}}{s}O$, while the output value is *x_k_*_+1_(2), called target velocity. A mode called Singer acceleration model is used to calculate the differential signal [13]. The Kalman filter’s parameters are as follows:

(6) $$A=\left[\begin{array}{ccc} 1 & T & 0.5T^{2}\\ 0 & 1 & T\\ 0 & 0 & 1 \end{array}\right],B={\left[\begin{array}{ccc} \frac{1}{6}T^{3} & \frac{1}{2}T^{2} & T \end{array}\right]}^{T},C=\left[\begin{array}{ccc} 1 & 0 & 0 \end{array}\right]$$

The process variance *Q_k_* is defined as to $Q_{k}=B\times B^{T}\times \sigma_{w}$, where $\sigma_{w}$ is variance of the observed value. The measurement variance *R_k_* is defined as to $\sigma_{v}$, which is the variance of sensor noise. The solution of the Kalman filter is below:

(7) $${\hat{x}}_{k+1}=(A-K_{k+1}CA){\hat{x}}_{k}+K_{k+1}y_{k}$$

The gain *K_k_*_+1_ of the Kalman filter can be obtained from the Ricatti equation if the matrices A, B and C are time-invariant and known. The reconstructive characteristic of the Kalman filter in the frequency domain can be described by Equation (8):

(8) $$\phi ={\left(ZI-A+K_{k+1}CA\right)}^{-1}K_{k+1}$$

The sampling frequency of the CCD is 50 Hz. The time delay *T*_0_ is 0.06 s. The open-loop transfer function without feed forward control is shown below:

(9) $$\frac{e^{-T_{0}s}}{s}C(s)P(s)\approx \frac{K_{p}(K_{I}s+1)}{s}\frac{e^{-T_{0}s}}{s}$$

For the feedback system to be robust, a gain margin larger than 6 dB and a phase margin larger than 35 degrees is usually specified [16], so the PI controller parameters can be obtained as *Kp* = 0.07096/$T_{0}^{2}$, *K_I_* = 7.1541*T*_0_.

The open-loop transfer function in Figure 2 is given by Equation (10):

(10) $$S_{open}=\frac{Q(s)+C(s)}{1-Q(s)P(s)\frac{e^{-T_{1}s}}{s}}P(s)\frac{e^{-T_{0}s}}{s}$$

The requirements of the closed-loop system with feedforward control need to meet a phase margin larger than 35° of the open-loop transfer function, so we have:

(11) $$\arg\left[S_{open}(s){|}_{s=j{w^{\prime }}_{c}}\right]+180^{o}\geq 35^{o}$$

From the above equation, we can obtain $T_{f}\geq 0.195$. The bandwidth of the filter is limited to about 1/(0.195×2π)=0.814Hz.

## 4. Performance Analysis

The sampling time is *T* = 0.02, the measurement variance is $\sigma_{v}=0.1$ and the process variance is $\sigma_{w}=100$. Therefore, a Kalman filter with a bandwidth of 0.99 Hz is preferable for the control system, since the phase margin (Pm) and the gain margin (Gm) of the open-loop transfer Equation (4) are 40.3° at the frequency of 1.33 Hz and 8.71 dB at the frequency of 3.69 Hz, respectively. The sensitivity function illustrated in Figure 3 is:

(12) $$S_{SF}=\frac{1-Q(s)P(s)\frac{e^{-T_{1}s}}{s}}{1+C(s)P(s)\frac{e^{-T_{0}s}}{s}+\frac{1}{s}Q(s)P(s)(e^{-T_{0}s}-e^{-T_{1}s})}$$

The sensitivity function without feedforward control is shown below:

(13) $${S^{\prime }}_{SF}=\frac{e^{-T_{0}s}}{1+C(s)P(s)e^{-T_{0}s}}$$

**Figure 6:**
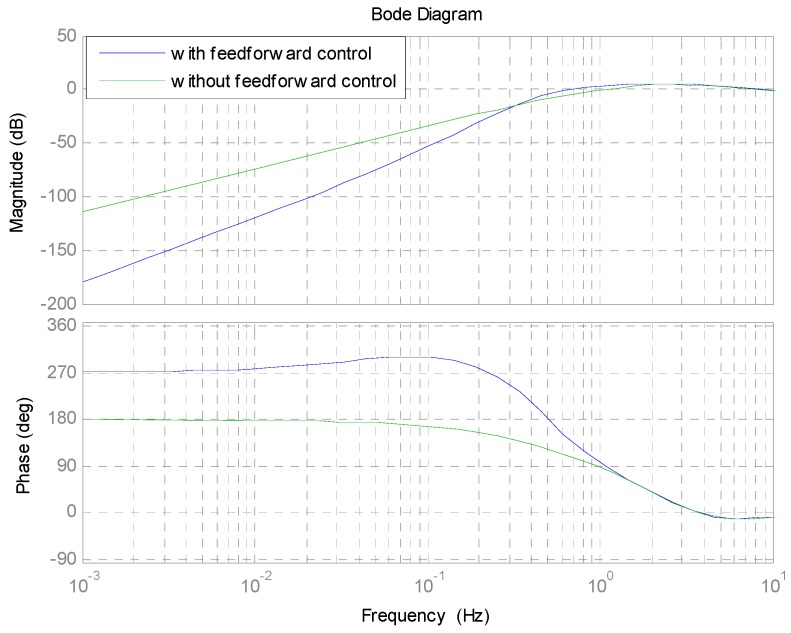
The sensitivity function response.

Based on the aforementioned considerations and design, the responses of the sensitivity Equations (12) and (13) are shown in Figure 6. A large attenuation, about one tenth less than that with only feedback loop control, is achieved in the low-frequency region with the feedforward controller in Figure 6. However, the attenuation in the middle-frequency range is magnified from 0.3 Hz to about 1.4 Hz, a little bigger than that achieved with the only feedback control. This is due to the amplification provided by the Kalman filter.

## 5. Simulations and Experiments

Verification of this control scheme was performed through simulations and experiments.

### 5.1. Simulations

**Figure 7:**
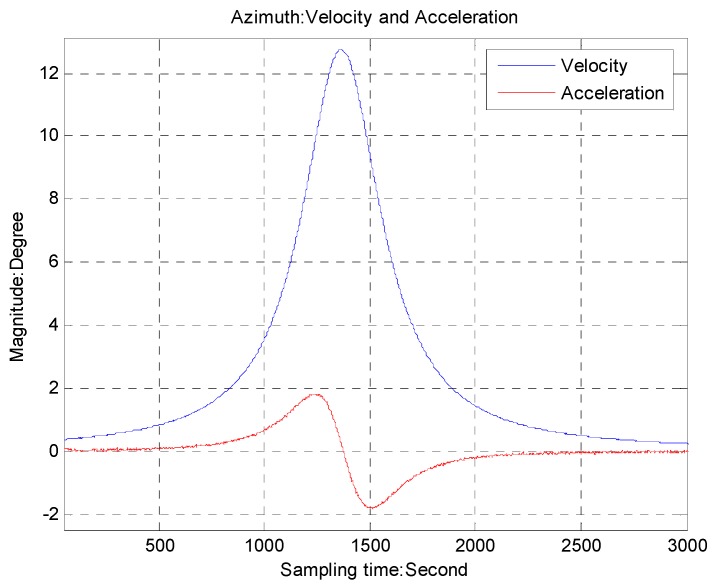
The simulative target velocity and acceleration.

**Figure 8:**
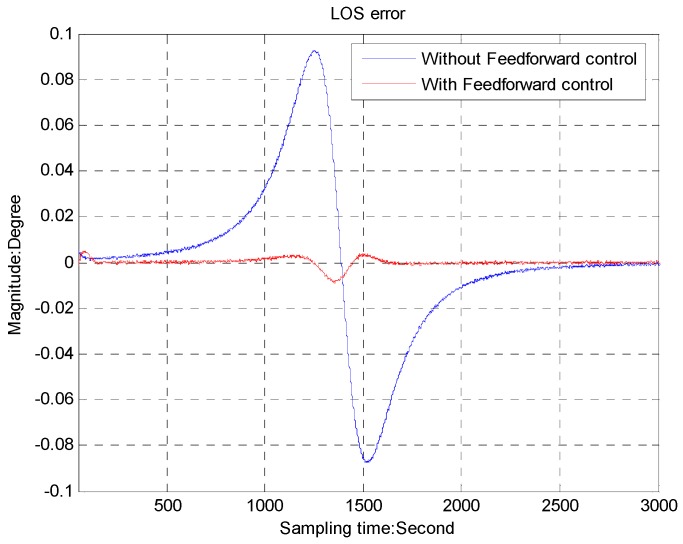
LOS error for the simulative target.

The sampling frequency of the CCD is 50 Hz. The precision of the rate sensor is about 0.01°/s. The target trajectory is emulated by the function *θ* = *a*tan(0.22*t* − 6.0), which has the maximum velocity 12.4°/s and the maximum acceleration 1.9°/s shown in Figure 7. This simulation only presents a single axis of the azimuth due to the similarity. The LOS error with and without feedforward control is compared in Figure 8. We can see that the maximum LOS error with the proposed method is 0.01°, about one-ninth of that with only feedback control.

### 5.2. Experiments

**Figure 9:**
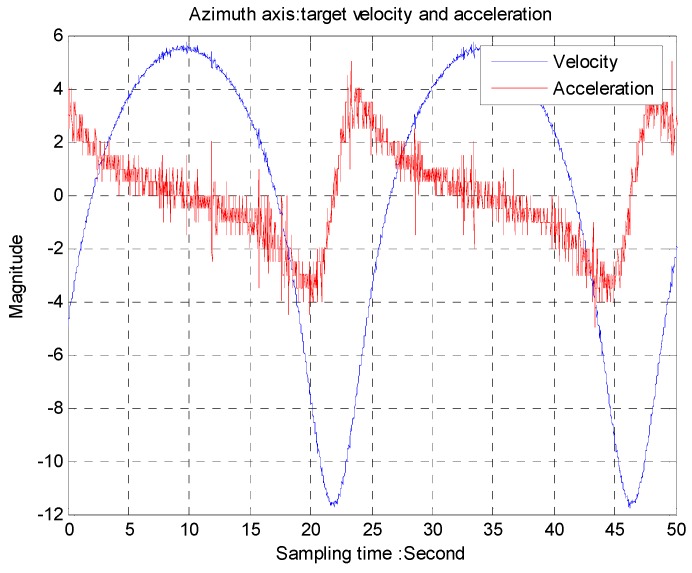
Azimuth axis: target velocity and acceleration.

**Figure 10:**
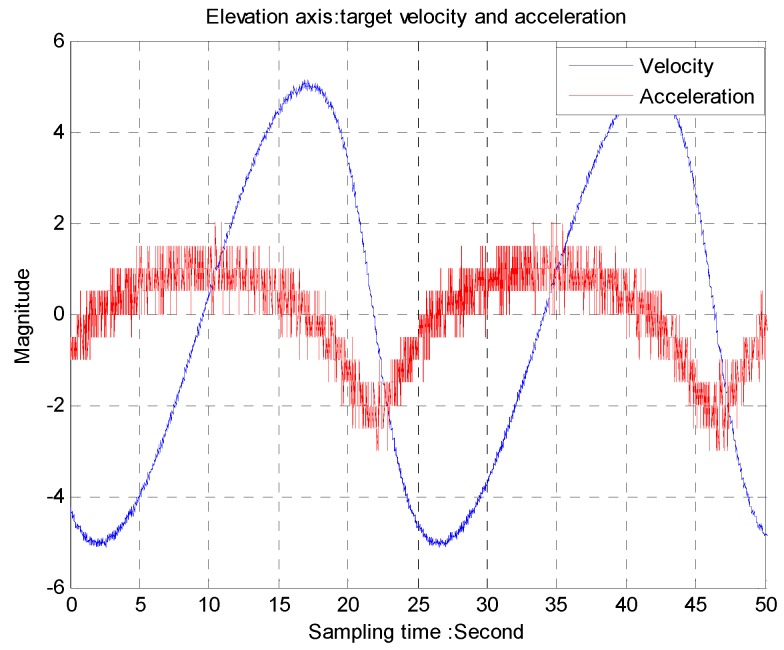
Elevation axis: target velocity and acceleration.

The trajectory of a moving target is provided by dynamic moving target simulators. A two-axis gimbal system with a 50 Hz CCD as a tracker is used to track the moving target. The angular speed from the gyro is shown in Figure 9 while acceleration is differentiated by the gyro as shown in Figure 10. The period of the trajectory is about 22 s, equivalent to 0.048 Hz.

**Figure 11:**
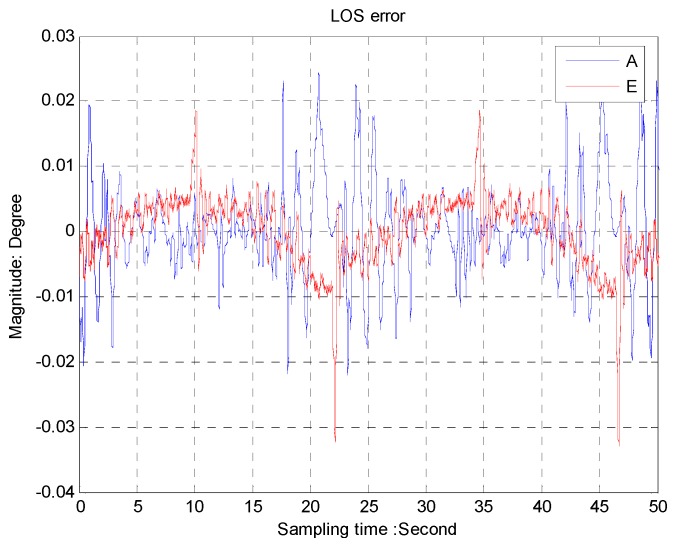
The LOS error with feed forward control.

**Figure 12:**
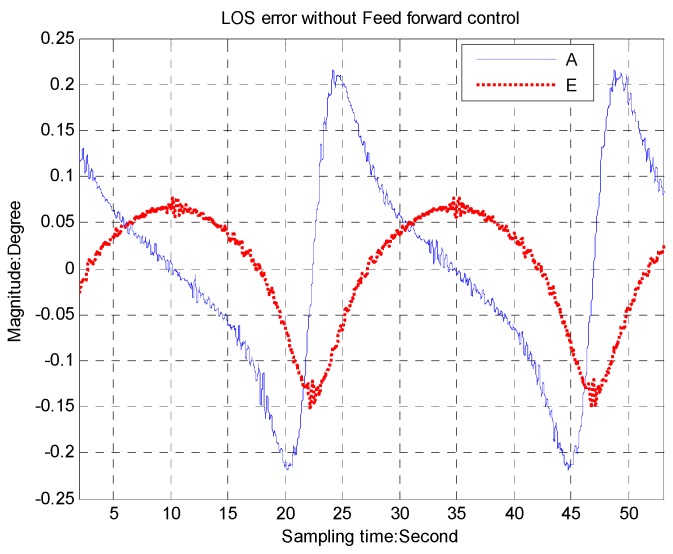
The LOS error without feed forward control.

The LOS errors with the feed forward control, shown in Figure 11, are about 0.02° for the azimuth axis and 0.01° for the elevation axis, while we can see in Figure 12 that the LOS errors are about 0.2° for the azimuth axis and 0.12° for the elevation axis, respectively, without the feed forward control.

Friction deteriorates tracking performance [17], resulting in a “not smooth error”, especially if the gimbal speed is low or approximates zero. It is thus important to compensate friction for a precision control system.

## 6. Conclusions

A feed forward control based on sensor fusion for a CCD-based tracking loop is proposed to reduce some error-related time delays. In this paper, we focus on the implementation of the feed forward control, the optimization of the control parameters and the analysis of the closed-loop stability. Experiments verify the technique proposed here effectively enhances the closed-loop performance in comparison with the classical control mode. There are some other topics not covered in this paper. The first one is how to optimize the control parameters in an all-round manner rather than in an independent way; second, it is very challenging to investigate acceleration feed forward [18] to improve performance; last but not least, some experiments are required to verify this method for an inertial stabilization platform [19].

## Figures and Tables

**Table 1:** CCD parameters.

**Frame Frequency**	50 Hz
**Pixels**	640 × 512
**Pixel Size**	5.5 µm
**Focus Length**	30–450 mm
